# Cost-effectiveness of screening of coronary artery disease in patients with type 2 DIABetes at a very high cardiovascular risk (SCADIAB study) rational and design

**DOI:** 10.1186/s12933-021-01253-2

**Published:** 2021-03-13

**Authors:** Kamel Mohammedi, Nathalie Préaubert, Tanguy Cariou, Vincent Rigalleau, Ninon Foussard, Laurent Piazza, Céline Bairras-Martin, Thierry Couffinhal, Julien Bezin, Antoine Benard

**Affiliations:** 1 Department of Endocrinology, Diabetes and Nutrition, Bordeaux University Hospital, Hôpital Haut-Lévêque, Avenue de Magellan, 33604 Pessac Cedex, France; 2 Faculty of Medicine, University of Bordeaux, Bordeaux, France; 3 INSERM Unit 1034, Biology of Cardiovascular Diseases, Pessac, France; 4 Health Economics Unit, Clinical Research Department, Bordeaux University Hospital, Talence, France; 5 Clinical Epidemiology Unit (USMR), CIC-EC 14-01, Bordeaux University Hospital, Bordeaux, France; 6 Internal Promotion Department, Bordeaux University Hospital, DCRI, Talence, France; 7 Department of Cardiology, Bordeaux University Hospital, Hôpital Haut-Lévêque, Bordeaux, Pessac France; 8 INSERM, Bordeaux Population Health Research Center, U1219, Team Pharmacoepidemiology, Bordeaux, France; 9 Department of Pharmacology, Bordeaux University Hospital, Bordeaux, France; 10 INSERM, Bordeaux Population Health Research Center, U1219, Team EMOS0, Bordeaux, France

**Keywords:** Type 2 diabetes, Coronary artery disease, Cardiovascular risk, Major adverse cardiac events, Screening, Real-world evidence study, Cost-effectiveness, Economic impact

## Abstract

**Background:**

Screening for coronary artery disease (CAD) remains broadly performed in patients with type 2 diabetes (T2DM), although the lack of evidence. We conduct a real-world evidence (RWE) study to assess the risk of major clinical outcomes and economic impact of routine CAD screening in T2DM individuals at a very high cardiovascular risk.

**Methods:**

SCADIAB is a comparative nationwide cohort study using data from the French National Health Data System. The main inclusion criteria are: age ≥ 40 years, DT2 diagnosed for ≥ 7 years, with ≥ 2 additional cardiovascular risk factors plus a history of microvascular or macrovascular disease, except CAD. We estimated ≥ 90,000 eligible participants for our study. Data will be extracted from 01/01/2008 to 31/12/2019. Eligible participants will be identified during a first 7-year selection period (2008–2015). Each participant will be assigned either in experimental (CAD screening procedure during the selection period) or control group (no CAD screening) on 01/01/2015, and followed for 5 years. The primary endpoint is the incremental cost per life year saved over 5 years in CAD screening group *versus* no CAD screening. The main secondary endpoints are: total 5-year direct costs of each strategy; incidence of major cardiovascular (acute coronary syndrome, hospitalization for heart failure, coronary revascularization or all-cause death), cerebrovascular (hospitalization for transient ischemic attack, stroke, or carotid revascularization) and lower-limb events (peripheral artery disease, ischemic diabetic foot, lower-limb revascularization or amputation); and the budget impact for the French Insurance system to promote the cost-effective strategy. Analyses will be adjusted for a high-dimension propensity score taking into account known and unknown confounders. SCADIAB has been funded by the French Ministry of Health and the protocol has been approved by the French ethic authorities. Data management and analyses will start in the second half of 2021.

**Discussion:**

SCADIAB is a large and contemporary RWE study that will assess the economic and clinical impacts of routine CAD screening in T2DM people at a very high cardiovascular risk. It will also evaluate the clinical practice regarding CAD screening and help to make future recommendations and optimize the use of health care resources.

*Trial registration* ClinicalTrials.gov Identifier: NCT04534530 (https://clinicaltrials.gov/ct2/show/NCT04534530)

## Background

Type 2 Diabetes mellitus (T2DM) is a major public health problem, responsible for a wide range of clinical, economic and societal issues [1–4]. Despite the substantial improvement in cardiovascular prognosis observed in recent decades, cardiovascular disease, essentially coronary artery disease (CAD), remains the leading cause of death [5]. CAD is often severe and silent in patients with T2DM [6–10], which may encourage a systematic and broad screening in asymptomatic individuals. Previous randomized controlled trial (RCT) did not provide evidence that routine screening for silent CAD may reduce the incidence of major cardiovascular events and death in people with T2DM [11–15]. Only one previous study showed that screening for silent CAD, compared with no screening, was associated with a reduced risk of minor cardiovascular events in T2DM patients with at least 2 cardiovascular risk factors [16]. However, the results of this monocentric and small sized study have never been corroborated by subsequent RCTs, although larger, multicentric and testing hard and validated clinical outcomes (major cardiovascular events and all-cause death) (Table 1). The DIAD (Detection of Ischemia in Asymptomatic Diabetics) study showed a comparable 4.8-year incidence of major cardiovascular event (a composite of cardiovascular death or non-fatal myocardial infarction) in 1123 T2DM participants who experienced CAD screening compared with those who did not [11]. Similar findings were reported in further large multicenter RCTs and a meta-analysis [12–15]. Nevertheless, some uncertainties remain in terms of benefit of CAD screening strategy in T2DM patients at very high cardiovascular risk, particularly those with peripheral atherosclerosis, chronic kidney disease (CKD) or any microvascular disease.

**Table 1:** Randomized controlled trials evaluating the effect of systematic screening of coronary artery disease in the risk of major cardiovascular adverse events in people with diabetes

Study	Population	Inclusion period	Follow-up (years)	Primary outcome	Sample size	Number of events	Expected incidence (%/year)	Observed incidence (%/year)	Hazard ratio (95% CI)
Faglia et al. [16]	T2DM Age 46–75 years +$\ge$ 2 CV risk factors	1998–1999	4.5	Major (cardiac death or MI) or minor (resting and effort angina) event	141	19	3	3	-
DIAD [11]	T2DM Age 50–70 years	2000–2002	4.8	Non-fatal MI or CV death	1123	32	1—2	0.6	0.88 (0.44–1.88)
DYNAMIT [12]	DT2M Age 55–75 years +$\ge$ 2 CV risk factors	2000–2003	3.5	All-cause death, non-fatal MI, non-fatal stroke, HH requiring hospitalization or visit to emergency department	631	54	5.5	2.4	1.00 (0.59–1.71)
FACTOR-64 [13]	T2DM + T1DM Men $\ge$ 50, Women $\ge$ 55 years diabetes duration $\ge$ 3 years or Men $\ge$ 40 years, Women $\ge$ 45y + Diabetes duration $\ge$ 5 years	2007–2013	4.0	All-cause death, nonfatal MI, or hospitalization for unstable angina	900	62	8	1.7	0.80 (0.49–1.32)
DADDY-D [14]	T2DM Age 50–70 years 10-year CV risk score ≥ 10%	2007–2012	3.6	Non-fatal MI or CV death	520	26	2.6	1.4	0.85 (0.39–1.83)

*T2DM* type 2 diabetes mellitus, *T1DM* type 1 diabetes mellitus, *CV* cardiovascular, *MI* myocardial infarction, *HH* heart failure

Despite this lack of evidence, most of guidelines recommend systematic screening for silent CAD in asymptomatic individuals with diabetes and high or very high cardiovascular risk [17–20]. Hence, a majority of physicians practice routine screening for silent CAD in patients with diabetes. We conducted a preliminary survey (unpublished data) in France in 2019 to determine the practices of 605 physicians in terms of screening for silent CAD in T2DM patients at a very high cardiovascular risk. A majority of participants (80% of cardiologists and 69% of diabetologists) reported a routine CAD screening practice with a sustained frequency: once a year (42%), once/2 years (20%), and once/3 years (33%).

A routine CAD screening induces high healthcare expenses as it leads to invasive investigations, endovascular and surgical revascularizations as well as intensification of pharmacological therapies. To the best of our knowledge, the cost-effectiveness of routine CAD screening has not been evaluated prospectively in population with T2DM and a very high cardiovascular risk. Only two economic studies, based on Markov models, have addressed this question by comparing different strategies in Japanese and American populations: no screening, screening (with stress echocardiography, myocardial scintigraphy coupled with stress test, or stress electrocardiogram) [21, 22]. Their results were very limited by model’s hypothesis; uncertainty surrounding epidemiological and utilities data used; and costs of care, which may differ greatly across countries. In this context of discrepancy between lack of evidence and current guidelines and clinical practice, we aim to estimate the effectiveness and cost-effectiveness of systematic CAD screening in patients with T2DM and a very high cardiovascular risk.

## Methods

### Study overview and design

The Cost-effectiveness of Screening of Coronary Artery disease in patients with type 2 DIABetes at a very high cardiovascular risk (SCADIAB) study is a retrospective and comparative real-world evidence (RWE) cohort study using data from the French National Health Data System (SNDS, *Système National des Données de Santé*), a claim database encompassing 98,8% of the whole French population [23].

### Study population

#### Inclusion criteria

Eligible participants must meet all of the following criteria:

- Age = 40 years or older,
- Affiliation to the general health insurance scheme in France,
- Diagnosis of T2DM,
- Duration of T2DM ≥ 7 years,
- Two or more additional cardiovascular risk factors (obesity, hypertension, hypercholesterolemia, or tobacco smoking using the chronic obstructive pulmonary disease (COPD) as a proxy),
- At least one microvascular or macrovascular disease: carotid stenosis, transient ischemic attack (TIA), stroke, lower-limb peripheral artery disease (PAD), CKD, severe diabetic retinopathy with requirement of laser photocoagulation, or peripheral or autonomic diabetic neuropathy.

Diagnosis will be determined based on the list of 100% health insurance coverage for chronic diseases in France (including T2DM, hypertension, TIA, stroke, PAD, CKD, COPD), the history of diseases of interest or surgery (coronary, carotid or lower-limb revascularization, bariatric surgery, kidney transplantation) according to the International Classification of Diseases Code Tenth Revision (ICD-10) (Additional file 1: Table S1), or at least 3 deliveries of one or more corresponding drugs as appropriate (antidiabetic, anti-obesity, antihypertensive or lipid-lowering drugs) over one year.

#### Exclusion criteria

One of the following:

- The presence of CAD (defined as a history of acute coronary syndrome, coronary revascularization, angina pectoris, or unstable angina).
- Any visit to an emergency department for chest pain resulting for admission to a cardiac intensive care unit.

### Recruitment of participants and follow‑up

**Fig. 1:**
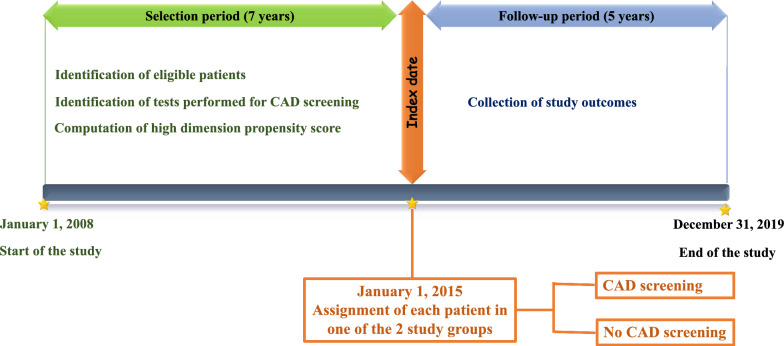
Study design

The data will be extracted from January 1, 2008 to December 31, 2019. A selection period will be defined by the 7 years (January 1, 2008 to December 31, 2014) preceding the index date. It will enable us to identify eligible patients and measure all the variables needed for the calculation of the high dimension propensity score (hdPS). Each participant will be assigned to one of the two study groups on the index date (January 1, 2015), and then followed up to December 31, 2019 (Fig. 1).

### Study groups and methods of CAD screening

#### Screening group

Individuals who had at least one screening exam (stress electrocardiogram test, stress echocardiography, myocardial scintigraphy coupled with stress test or pharmacological stimulation using adenosine, or coronary CT scan) (Table 2) during the selection period (2008–2015).

**Table 2:** Codes used to identify tests to screen coronary artery disease

Tests	Procedure codes
Stress test	DKRP004, EQRP002, EQRM001
Myocardial scintigraphy coupled with stress test or pharmacological stimulation using adenosine	DAQL001, DAQL010, DAQL011, DAQL009
Stress echocardiography	DAQM003, DZQM002, DBQM001
Coronary CT scan	ECQH010
Procedure codes: Codes of the French classification of medical procedure (CCAM)	

#### No-screening group

Participants who never experienced a screening exam as described above during the selection period (2008–2015), except resting electrocardiogram.

### Study endpoints

#### Primary endpoint

The incremental cost per life year saved over 5 years in CAD screening group *versus* no CAD screening.

### Secondary endpoints

#### Economic endpoints

(1) A cost-consequence analysis linking the total 5-year direct costs of each strategy (drugs, medical visits, hospitalizations, nursing visits, biological and radiological exams, technical exams, medical transports…) and the total number of major cardiovascular and renal events over 5 years; (2) the budget impact (in €) for the French Insurance system to promote the most cost-effective strategy between routine CAD screening and no screening; and (3) the total care consumption over the follow-up period.

#### Clinical endpoints

The effects of CAD screening (versus no CAD screening) in terms of major adverse cardiac events (the first occurrence of any component of the composite outcome, which comprise acute coronary syndrome, coronary revascularization, hospitalization for heart failure or all-cause death); major cerebrovascular events (the first occurrence either of stroke, hospitalization for TIA, or carotid revascularization); major adverse limb events (the first occurrence of any component of the composite outcome, comprising PAD, ischemic diabetic foot, lower-limb revascularization (angioplasty or surgery) or amputation); and CKD or end-stage kidney disease (ESKD, defined as requirement of any sustained renal replacement therapy or kidney transplantation). Each component of these composite endpoints will also be considered individually. Major cerebrovascular events, major adverse limb events and ESKD will be considered during follow-up among participants without a history of each appropriate condition at baseline. Clinical endpoints will be determined according to ICD-10 and codes of the French classification of medical procedure (CCAM) as presented in Additional file 2: Table S2.

Finally, SCADIAB study will also assess the frequency of routine CAD screening, expressed as the number of examinations performed per individual per year.

### Statistical considerations

#### Sample size estimation

Among 3.3 million people treated for diabetes mellitus in France, at least 3 million are estimated to have T2DM. Based on previous data, about 2.4 million T2DM individuals would be free of a history of CAD, 38% would have at least two cardiovascular risk factors, and a history of microvascular or macrovascular disease would be present in more than 10% of patients [24, 25]. Hence, we estimated that at least 90,000 patients would be eligible for our study.

### Statistical analysis plan

The data will be analysed by the biostatistician of the “Clinical Epidemiology Unit” of the University Hospital of Bordeaux (USMR). Analyses will be performed using SAS® software, version 9.4 or later (SAS Institute, Cary, NC, USA, http://www.sas.com) and all tests will be performed at the first-order error risk α = 5%. The flow-chart (CONSORT), as well as the characteristics of patients at inclusion (eligibility criteria, epidemiological, clinical, biological characteristics and treatment use) will be presented. Confounding factors will be taken into account through a hdPS score that will involve around 500 variables, according to the Bross formula [26]. A principal component analysis will then allow us to reduce the dimensionality to 30 components, and the goodness of fit of this hdPS score will be estimated by a graphical comparison of the score distribution and by standardized mean differences between the two study groups [27]. Comparisons between the two groups will be carried out with and without adjustment for the hdPS score, and the hypotheses of the different regression models chosen, will be systematically verified.

Regarding the analysis of the primary endpoint, a gross estimate of the incremental cost per life-year gained at 5 years will be conducted. The confidence interval (CI) of this incremental cost-effectiveness ratio will be estimated by boostrap (5000 iterations). The analysis will be performed as Intent to Treat. To investigate a possible relationship between the frequency of screening and survival, we will define four different subgroups: screening conducted at least once a year, less than once a year (and at least once every two years), less than once every two years (and at least once every three years), and only once during the follow-up period.

A cost-consequence analysis will be performed linking results of all direct costs (observed during the following period) and results on major cardiovascular events. A budget impact analysis will be conducted to determine the public expenses of the spread of a systematic screening for silent ischemic CAD.

Cardiovascular events will be expressed as numbers, cumulative incidence and incidence rates. Kaplan–Meier curves will be elaborated to plot the incidence of endpoints according to study groups (CAD screening versus no screening). Unadjusted comparisons between groups will be done using a log-rank test. Cox proportional hazards regression models will be computed to estimate Hazard ratios, with related 95% CIs, after adjustment for hdPS score.

### Study progress

The study protocol has been approved by the French ethic authorities (see “Declarations” section below). Procedures and contracts for access to the SNDS databases are in progress. Data management and analyses will start in the second half of 2021, and the main results will be published in 2022.

## Discussion

We will conduct a large RWE study to investigate the risk of major clinical outcomes and economic impact of routine CAD screening in individuals with T2DM and a very high risk for cardiovascular disease in France. We will perform a retrospective analysis using the electronic health record (EHR) data from the SNDS databases which represent almost the entire French population allowing us sufficient sample size to address the investigated question.

### Screening for silent CAD and risk of major cardiovascular events

Previous RCTs demonstrated no clinical benefit associated with routine CAD screening in asymptomatic T2DM patients, but population with a high cardiovascular burden and any organ vascular damage has not been investigated [11–14]. Also, the observed incidence of outcomes was much lower than expected in published RCTs evaluating the interest of routine CAD screening in individuals with T2DM (Table 1). The conduction of a new prospective RCT will be too expensive, time-consuming with difficulties for recruiting asymptomatic participants with a very high cardiovascular risk. Indeed, the DYNAMIT (Do You Need to Assess Myocardial Ischemia in Type-2 diabetes) study was stopped prematurely due to difficulties in recruitment of participants and a low incidence of cardiovascular events [12]. DYNAMIT included only one-fifth (631/3000) of the originally planned enrolment to detect a 20% relative risk reduction (RRR) in the primary endpoint (a composite of all-cause death, non-fatal myocardial infarction, non-fatal stroke, or heart failure requiring hospitalization or visit to emergency department) in people with T2DM at a high cardiovascular risk (aged 55 to 75 years with at least 2 other cardiovascular risk factors). A recent meta-analysis estimated that a large number of participants would be needed to demonstrate a potential benefit of the systematic CAD screening strategy to reduce the incidence of major cardiovascular events [15]. The optimal sample size for 20% RRR of major cardiac events should be 29,763 participants (19,548 for all-cause death). Therefore, a retrospective RWE study seems to be an appropriate approach to evaluate the long-term cost-effectiveness of systematic CAD screening strategy in T2DM subjects at a very high cardiovascular risk without known history of CAD. Of note, among 3.7 million people with diabetes in France, we estimate that at least 90,000 individuals will be eligible for our study in the SNDS database.

### Cost-effectiveness of routine CAD screening in patients with diabetes

Systematic CAD screening strategy leads to more invasive examination, especially coronary angiography, which is associated with increased risk of complications [11, 12]. Routine CAD screening encourages also revascularizations and intensification of pharmacological treatments, despite uncertainties in terms of related benefits. In the FACTOR-64 study, CAD screening was not associated with a significant reduction in major cardiovascular events despite intensive pharmacological control of cardiovascular risk factors (and some coronary revascularizations) in patients with a positive screening test, while individuals assigned to control group and those with a negative screening test had only conventional cardiovascular treatment goals [13]. Furthermore, BARDOT (Basel Asymptomatic high-Risk Diabetics' Outcome Trial) study showed that combined medical therapy and invasive strategy (coronary angiography followed or not by revascularization) for silent CAD, compared with medical treatment alone, was associated with reduced scintigraphic CAD progression, but no significant difference was observed in terms of hard clinical events in high risk T2DM patients [28]. Therefore, to avoid unjustified health expenses and misuse of collective resources associated with routine CAD screening, we conduct the SCADIAB study to assess the cost-effectiveness of this strategy in people with T2DM and a very high cardiovascular risk.

### Strengths and limitations

The key strength of our study is the collection of a comprehensive range of clinical features, cardiovascular procedures, history of pharmacological therapies or surgery, major events and survival status in the whole French population of patients with T2DM and a very high cardiovascular risk during an overall period of 12 years. SCADIAB findings will have a broader generalizability for all T2DM patients at a very high cardiovascular risk. Our study will provide a line of complementary evidence (further to RCTs’ findings) in terms of routine CAD screening in a contemporary cohort of patients with T2DM in real-world settings. SCADIAB will be more economical and time efficient than RCT, but a number of intrinsic limitations need to be acknowledged. The retrospective and non-randomized design of our study is subject to bias (selection, information, detection) and confounding factors. To limit selection bias, our study population will be rigorously defined using pre-specified inclusion criteria (T2DM for at least 7 years, with at least 2 cardiovascular risk factors and one or more organ damage) extracted from reliable and valid SNDS database. The 7-year selection period leading to assignment of each individual to one of the two study arms (CAD screening or absence of CAD screening) will limit information bias. We will use validated algorithms to identify inclusion criteria and endpoints [29–31]. Also, the cardiovascular investigation required for study arms assignment (stress test, scintigraphy, stress echocardiography…) will be recorded exhaustively in the SNDS database. We believe that the detection bias will be limited in our study as we have access to the SNDS database covering the whole French population including death registry. Finally, we will use the hdPS method to control measurable confounding factors as well as unknown or unmeasurable ones [32–34].

## Conclusions

SCADIAB study is the first investigation of the cost-effectiveness of CAD screening strategy in T2DM patients with a very high cardiovascular risk. It will evaluate the economic impact and clinical benefits of routine CAD screening in this population. It will also evaluate the clinical practice regarding CAD screening. SCADIAB will provide essential information for payers, clinicians, and scientific societies in terms of long-term efficiency of systematic CAD screening in a large T2DM population. Our study will also measure the economic performance of CAD screening in a real-world setting, with an accurate comparison with no CAD screening strategy. The SCADIAB findings will help to optimize the use of health care resources and guide clinical decision-making and future recommendations.

## Supplementary Information

**Additional file 1: Table S1.** Codes used to identify inclusion and exclusion criteria.

**Additional file 2: Table S2.** Codes used to identify study endpoints.

## Data Availability

Not applicable.

## Abbreviations

BARDOT: Basel Asymptomatic high-Risk Diabetics' Outcome Trial
CAD: Coronary Artery Disease
CCAM: *Classification Commune des Actes Médicaux* (The French classification of medical procedures)
CI: Confidence Interval
CKD: Chronic Kidney Disease
COPD: Chronic Obstructive Pulmonary Disease
DYNAMIT: Do You Need to Assess Myocardial Ischemia in Type-2 diabetes
EHR: Electronic Health Record
ESKD: End-Stage Kidney Disease
HDPS: High-Dimension Propensity Score
ICD-10: International Classification of Diseases Code Tenth Revision
PAD: Peripheral Artery Disease
RCT: Randomized Controlled Trial
RRR: Relative Risk Reduction
RWE: Real-World Evidence
SCADIAB: Cost-effectiveness of Screening of Coronary Artery disease in patients with type 2 DIABetes at a very high cardiovascular risk
SNDS: *Système National des Données de Santé* (The French National Health Data System)
T2DM: Type 2 Diabetes Mellitus
TIA: Transient Ischemic Attack
