# Supplementary material for: Cost-effectiveness of screening of coronary artery disease in patients with type 2 DIABetes at a very high cardiovascular risk (SCADIAB study) rational and design
Source: Cardiovasc Diabetol. 2021 Mar 13;20:63. doi: 10.1186/s12933-021-01253-2 (PMC7955624; doi:10.1186/s12933-021-01253-2)
Supplement: Supplementary file 1 — Additional file 1: Table S1. Codes used to identify inclusion and exclusion criteria. [file 12933_2021_1253_MOESM1_ESM.docx]

**Additional file 1: Table S1. Codes used to identify inclusion and exclusion criteria.**

| **Criteria** | **ICD-10 Codes** | **ATC Codes** | **Procedure codes** |
| --- | --- | --- | --- |
| Type 2 diabetes | E11 | A10B |  |
| Type 1 diabetes | E10 |  |  |
| Gestational diabetes | O24 |  |  |
| Other types of diabetes | E13 |  |  |
| Obesity | E66 | A08 |  |
| Hypertension | I10-I13, I15, I674 | C03, C07, C08, C09, C02AC, C02CA, C02AB, C02DC, C02L |  |
| Lipid-lowering drugs |  | C10AA, C10AX09, C10B |  |
| Chronic obstructive pulmonary disease | J44 |  |  |
| Carotid stenosis | I65.2 |  | ECAF003, ECAF004, ECLF004  EBAF004, EBAF010  EBFA005, EBFA003, EBFA010  EBCA015, EBCA004, EBCA008, EBCA001, EBCA017, EBEA005, EBEA003  EBAF003, EBAF001, EBAF005, EBAF006  EBCA010, EBCA011, EBCA002, EBCA013  EAAF004, EAAF002, EAAF901, EAAF900  EBAF013, EBAF014, EAAF903, EAAF902  EBCA014, EBCA005, EBEA002, EBEA004 |
| Transient ischemic attack | G45  (except G454) |  |  |
| Stroke | I61-I64 |  |  |
| Lower-limb peripheral artery disease | I70, I73, I74 |  |  |
| Diabetic nephropathy | (E10-E14).2 associated with N08.3 |  |  |
| Chronic kidney disease, end-stage kidney disease | N18 |  | HGPC005, HPGA001, HPJP001, HPKA002, HPKB001, HPKC014, HPLA005, HPLB004, HPLC035, HPMP002, HPPA004, HPPP002, JVJB001, JVJB002, JVJF002, JVJF003, JVJF004, JVJF005, JVJF006, JVJF007, JVJF008, JVQF001, JVQF007, JVQP009, JVRP004, JVRP007, JVRP008, YYYY007 |
| Severe diabetic retinopathy with requirement of laser photocoagulation | (E10-E14).3 associated with H36.0 |  | BGNP004, BGNP003, BGNP007, BGNP008, BGNP001, BGMA002, BGMA001 |
| Peripheral or autonomic diabetic neuropathy | (E10-E14).4 associated with G73.0, G59.0, G99.0 or G63.2 |  |  |
| Coronary artery disease | I20-I25 |  | DDAA002, DDAF001, DDAF003, DDAF004, DDAF006-DDAF010, DDFF001, DDFF002, DDLF001, DDPF002, DDSF001, YYYY082, DDMA002-DDMA009, DDMA011-DDMA013, DDMA015-DDMA038, ENFA003, EPFA006, DDQH006, DDQH009, DDQH015 |
| Chest pain | R07.2-R07.4 |  |  |
| ICD-10: International Classification of Diseases Code Tenth Revision  ACT: Anatomical therapeutic chemical classification system  Procedure codes: Codes of the French classification of medical procedure (CCAM) | | | |
