# Supplementary material for: Cost-effectiveness of screening of coronary artery disease in patients with type 2 DIABetes at a very high cardiovascular risk (SCADIAB study) rational and design
Source: Cardiovasc Diabetol. 2021 Mar 13;20:63. doi: 10.1186/s12933-021-01253-2 (PMC7955624; doi:10.1186/s12933-021-01253-2)
Supplement: Supplementary file 2 — Additional file 2: Table S2. Codes used to identify study endpoints. [file 12933_2021_1253_MOESM2_ESM.docx]

Additional file 2: Table S2. Codes used to identify study endpoints

| **Endpoints** | **ICD-10 Codes** | **Procedure codes** |
| --- | --- | --- |
| Major adverse cardiac events |  |  |
| Acute coronary syndrome | I200, I21, I24 |  |
| Heart failure | I50, I110, I130, I132, I139, K761 |  |
| Coronary revascularization |  | DDAA002, DDAF001, DDAF003, DDAF004, DDAF006-DDAF010, DDFF001, DDFF002, DDLF001, DDPF002, DDSF001, YYYY082, DDMA002-DDMA009, DDMA011-DDMA013, DDMA015-DDMA038, ENFA003, EPFA006, DDQH006, DDQH009, DDQH015 |
| Major cerebrovascular events |  |  |
| Transient ischemic attack | G45 (except G454) |  |
| Stroke | I61-I64 |  |
| Carotid revascularization |  | EBAF003, EBAF001, EBAF005, EBAF006  EBCA010, EBCA011, EBCA002, EBCA013  EAAF004, EAAF002, EAAF901, EAAF900  EBAF013, EBAF014, EAAF903, EAAF902  EBCA014, EBCA005, EBEA002, EBEA004 |
| Major adverse limb events |  |  |
| Lower-limb peripheral artery disease | (E10-E14).5, L97 |  |
| Ischemic diabetic foot | E145, E147, I70.21, L97 |  |
| Lower-extremity amputation (toe, transmetatarsal, transtibial, transfemoral) |  | NZFA007, NZFA002, NZFA009, NZFA005, NZFA013, NZFA010, NZFA004 |
| Lower-limb revascularization |  | EEAF003, EEAF004, EEAF005, EEAF002, EEAF001, EEAF006  EENF002, EENF001, EEPF002, EEPF001, EEJF001, EEFA004, EEFA002, EEFA001, EEFA003  EECA007, EECA006, EDCA005, EDCA004, EECA002, EECA001, EECA003, EECA008, EECA010, EECA005, EECA012 |
| Chronic kidney disease, end-stage kidney disease | N18 | HGPC005, HPGA001, HPJP001, HPKA002, HPKB001, HPKC014, HPLA005, HPLB004, HPLC035, HPMP002, HPPA004, HPPP002, JVJB001, JVJB002, JVJF002, JVJF003, JVJF004, JVJF005, JVJF006, JVJF007, JVJF008, JVQF001, JVQF007, JVQP009, JVRP004, JVRP007, JVRP008, YYYY007 |
| ICD-10: International Classification of Diseases Code Tenth Revision  Procedure codes: Codes of the French classification of medical procedure (CCAM) | | |
